# Heating ability of magnetic nanoparticles with cubic and combined anisotropy

**DOI:** 10.3762/bjnano.10.29

**Published:** 2019-01-29

**Authors:** Nikolai A Usov, Mikhail S Nesmeyanov, Elizaveta M Gubanova, Natalia B Epshtein

**Affiliations:** 1 National University of Science and Technology «MISIS», 119049, Moscow, Russia; 2 Pushkov Institute of Terrestrial Magnetism, Ionosphere and Radio Wave Propagation, Russian Academy of Sciences, IZMIRAN, 108480, Troitsk, Moscow, Russia; 3 National Research Nuclear University “MEPhI”, 115409, Moscow, Russia

**Keywords:** fractal clusters, magnetite nanoparticles, magneto–dipole interaction, numerical simulation, specific absorption rate

## Abstract

The low frequency hysteresis loops and specific absorption rate (SAR) of assemblies of magnetite nanoparticles with cubic anisotropy are calculated in the diameter range of *D* = 20–60 nm taking into account both thermal fluctuations of the particle magnetic moments and strong magneto–dipole interaction in assemblies of fractal-like clusters of nanoparticles. Similar calculations are also performed for assemblies of slightly elongated magnetite nanoparticles having combined magnetic anisotropy. A substantial dependence of the SAR on the nanoparticle diameter is obtained for all cases investigated. Due to the influence of the magneto–dipole interaction, the SAR of fractal clusters of nanoparticles decreases considerably in comparison with that for weakly interacting nanoparticles. However, the ability of magnetic nanoparticle assemblies to generate heat can be improved if the nanoparticles are covered by nonmagnetic shells of appreciable thickness.

## Introduction

Magnetic hyperthermia [1–3] is a promising therapeutic method that can be used in combination with chemotherapy or radiotherapy for cancer treatment. Iron oxide nanoparticles are among the materials most popular for application in biomedicine due to their biocompatibility, biodegradability [4] and sufficiently high saturation magnetization [5]. However, only nanoparticles with a high specific absorption rate (SAR) in an alternating external magnetic field are suitable for magnetic hyperthermia. Therefore, a significant number of recent experimental studies [6–14] have been devoted to the development of advanced methods for the synthesis of iron oxide nanoparticles and measurement of their SAR under various conditions. It should be noted that in theoretical SAR calculations [15–22] the assemblies of magnetic nanoparticles with uniaxial magnetic anisotropy have mostly been studied. Meanwhile, perfect iron oxide nanoparticles of spherical shape should have cubic-type magnetic anisotropy [5]. However, to describe the existing experimental data properly one has also to take into account the possible perturbation of the nanoparticle shape. A deviation from the nanoparticle shape in a first approximation can be described as small particle elongation in a direction random with respect to the direction of the cubic easy anisotropy axes. For a slightly elongated nanoparticle of a soft magnetic type, the shape anisotropy energy may have an appreciable contribution to the total particle energy. As a result, the nanoparticles having shape perturbation possess combined magnetic anisotropy [23].

In this paper the low frequency hysteresis loops and the SAR of magnetite nanoparticles with cubic and combined magnetic anisotropy have been calculated using numerical simulations. We take into account the influence of thermal agitation of particle magnetic moments at a room temperature and the effect of mutual magneto–dipole interaction between the nanoparticles on the assembly behavior. The aim of this paper is to estimate the SAR of magnetite nanoparticles in biological media to quantitatively predict their heating efficiency in magnetic nanoparticle hyperthermia. In this respect we would like to stress that the behavior of an assembly of magnetic nanoparticles in viscous liquids and biological media is different [2–3]. It has been proved recently [13,24–25] that in biological media the magnetic nanoparticles can agglomerate within the biological cells or in the intracellular environment. The dense clusters of the nanoparticles turn out to be tightly bound to the surrounding media, so that the rotation of the nanoparticles as a whole is greatly hindered. It is also important that the average distance between the centers of closest nanoparticles in the cluster is small, of the order of the particle diameter. Therefore, the strong magneto–dipole interaction within the clusters considerably affects the heating efficiency of the assembly [18–22,24–25]. In addition, in the calculations performed, the fractal nature [24–27] of magnetic clusters in biological media is taken into account.

The numerical simulations are carried out using the Landau–Lifshitz (LL) stochastic equation [22,28–31]. It is found that, similar to the case of interacting uniaxial nanoparticles [22], the strong magneto–dipole interaction considerably decreases the SAR of fractal clusters of magnetite nanoparticles. However, the dependence of the SAR on the mean nanoparticle diameter is retained, being less pronounced for strongly interacting nanoparticles. It is also important for clusters of magnetite nanoparticles with cubic and combined magnetic anisotropy that the maximal SAR values shift to larger particle diameters with respect to those for similar nanoparticles with uniaxial anisotropy [22]. This is attributed to a decreased value of effective energy barriers for particles with cubic or combined anisotropy. It has been previously shown for an assembly of uniaxial nanoparticles [22] that the existence of nonmagnetic shells of appreciable thickness at the nanoparticle surface considerably reduces the intensity of the magneto–dipole interaction within the cluster. A similar effect is also confirmed for clusters of nanoparticles with cubic or combined anisotropy. For example, “protein coronas” around the nanoparticles can keep them separated in a natural way. This effect can be used to improve the ability of magnetite nanoparticles to generate heat in an alternating external magnetic field.

According to the present calculations, for interacting magnetite nanoparticles with cubic or combined anisotropy, sufficiently high SAR values of the order of 250–350 W/g can be obtained for low values of magnetic field amplitudes, *H*_0_ = 50–100 Oe, at a typical frequency *f* = 300 kHz. This shows the substantial potential of these nanoparticles for application in magnetic nanoparticle hyperthermia.

## Numerical Simulation

It has been recently shown [22] that the technique based on the stochastic LL equation is preferable for investigation of the properties of interacting assemblies of superparamagnetic nanoparticles with uniaxial anisotropy. In the present manuscript the same approach is used to study the behavior of dense clusters of magnetite nanoparticles with cubic or combined anisotropy.

The stochastic LL equation [22,28–31] governs the dynamics of the unit magnetization vector 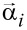 of the *i*th single-domain nanoparticle of the cluster

[1]
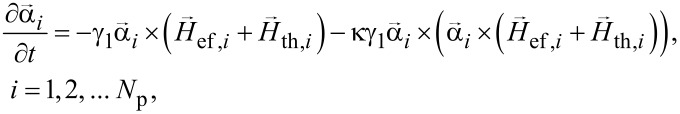


where γ is the gyromagnetic ratio, κ is phenomenological damping parameter, γ_1_ = γ/(1+κ^2^), 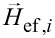 is the effective magnetic field and 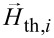 is the thermal field. The effective magnetic field acting on a separate nanoparticle can be calculated as a derivative of the total cluster energy

[2]
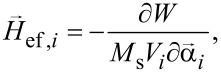


where *V_i_* is the volume of the *i*th nanoparticle. The total magnetic energy of the cluster, *W* = *W*_a_ + *W*_Z_ + *W*_m_, is a sum of the magnetic anisotropy energy, *W*_a_, the Zeeman energy, *W*_Z_, of the particles under an applied magnetic field, and the energy of the mutual magneto–dipole interaction of the particles, *W*_m_.

For nanoparticles with cubic type magnetic anisotropy, the magneto-crystalline anisotropy energy is given by

[3]
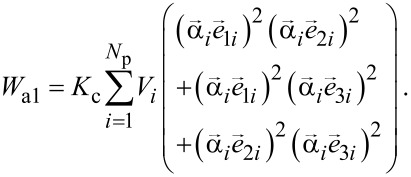


Here *K*_c_ is the cubic magnetic anisotropy constant, and (***e***_1_*_i_*, ***e***_2_*_i_*, ***e***_3_*_i_*) is a set of orthogonal unit vectors that determine an orientation of the *i*th nanoparticle of the cluster.

In this paper we also consider slightly elongated nanoparticles of spheroidal shape with semiaxis ratio *a*/*b* > 1. It is supposed that the semiaxis ratios of various nanoparticles of the cluster are randomly distributed within a small interval 1 < *a*/*b* < ξ_max_. For elongated nanoparticles, in addition to the magneto-crystalline anisotropy energy, Equation 3, there is also a shape anisotropy energy contribution

[4]
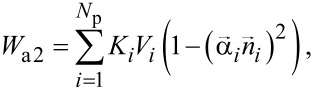


where *K_i_* is the shape anisotropy constant and ***n****_i_* is the unit vector along the direction of elongation of the *i*th nanoparticle. For the shape anisotropy constant one obtains [32]


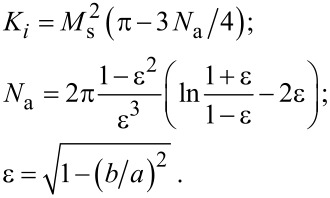


Here *N*_a_ is the demagnetizing factor along the long nanoparticle axis.

Next, the Zeeman energy of the cluster in an applied alternating magnetic field 
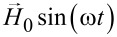
 is given by

[5]
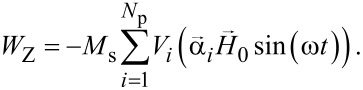


For nearly spherical, uniformly magnetized nanoparticles the magnetostatic energy of the cluster can be represented as the energy of the point interacting dipoles located at the particle centers ***r****_i_* within the cluster. Then the energy of magneto–dipole interaction is

[6]
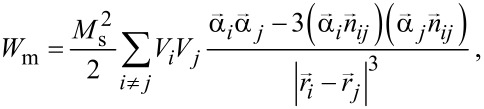


where ***n****_ij_* is the unit vector along the line connecting the centers of the *i*th and *j*th particles, respectively.

The thermal fields 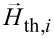 acting on various nanoparticles of the cluster are statistically independent, with the following statistical properties [28] of their components

[7]
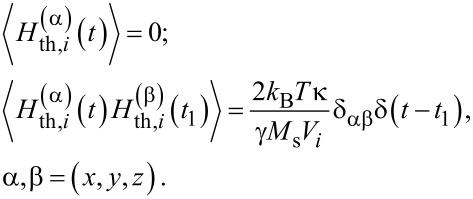


Here *k*_B_ is the Boltzmann constant, δ_αβ_ is the Kroneker symbol, and δ(*t*) is the delta function.

**Figure 1:**
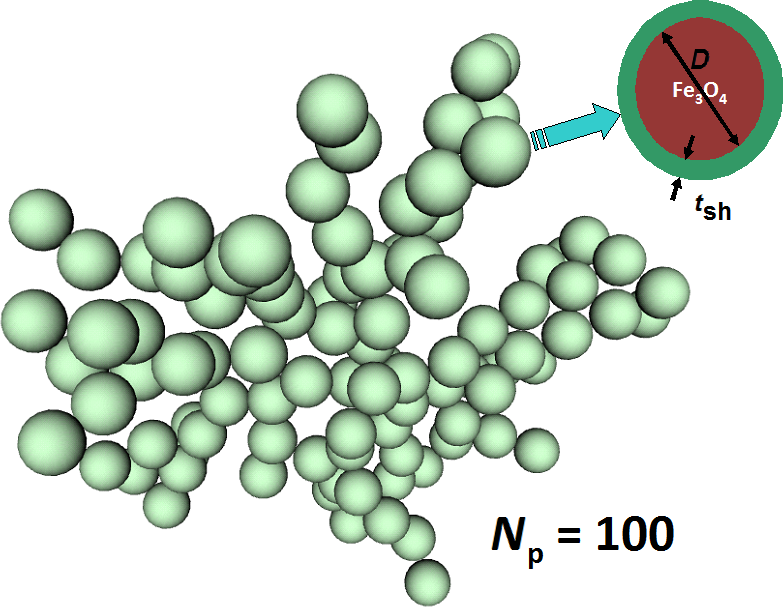
Geometry of fractal cluster of single-domain nanoparticles with fractal dimension *D*_f_ = 1.9 and prefactor *k*_f_ = 1.7. The inset shows an isolated magnetite nanoparticle of diameter *D* covered with a nonmagnetic shell of thickness *t*_sh_.

It is well known [33–34] that the geometrical structure of a fractal cluster of nanoparticles is characterized by the relation 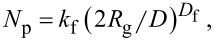 where *N*_p_ is the total number of the nanoparticles in the cluster, *D*_f_ is the fractal dimension, and *k*_f_ is the the fractal prefactor. The radius of gyration *R*_g_ is defined as the mean square of the distances between the particle centers and the geometrical center of mass of the cluster. Interestingly, in contrast to usual 3D clusters, the dimension *D*_f_ of a fractal cluster is typically a noninteger number. The fractal clusters with various fractal descriptors *D*_f_ and *k*_f_ were generated in this paper using the well known Filippov’s et al. algorithm [34]. Most of the calculations were performed for fractal clusters with *D*_f_ = 1.9 and *k*_f_ = 1.7 because these clusters are observed most often in biological media [24–25]. Similar results were obtained also for clusters with other fractal descriptors. As an example, Figure 1 shows the geometrical structure of fractal cluster with fractal descriptors *D*_f_ = 1.9 and *k*_f_ = 1.7 consisting of *N*_p_ = 100 single-domain nanoparticles.

The random space orientation of the *i*th spherical nanoparticle with cubic magnetic anisotropy is determined by a set of orthogonal unit vectors (***e***_1_*_i_*, ***e***_2_*_i_*, ***e***_3_*_i_*), *i* = 1, 2, …, *N*_p_. The nanoparticles of a fractal cluster are also characterized by the coordinates of their centers {***r****_i_*}, as well as by the diameter *D* and the nonmagnetic shell thickness *t*_sh_ which are supposed to be the same for all nanoparticles of the given cluster. For spheroidal nanoparticles with combined anisotropy the directions of the particle elongations ***n****_i_* are unit vectors randomly distributed in space – the semiaxis ratios ξ*_i_* = 2*a_i_*/*D* are random variables uniformly distributed within the interval 1 ≤ ξ*_i_* ≤ ξ_max_. Here *a_i_* is the long semiaxis of the *i*th nanoparticle, whereas its transverse semiaxis equals *D*/2. The nanoparticle elongations are supposed to be small, ξ_max_ ≤ 1.2, so that various spheroids are close to a sphere. The calculations show that in the limit *N_p_* >> 1 the averaged hysteresis loop of cluster assembly has a rather small dispersion being averaged over 30–40 independent realizations of random clusters with the fixed values of the initial parameters *D*, *t*_sh_, *N*_p_ and ξ_max_.

## Simulation Results

### Nanoparticles with cubic anisotropy

Let us first consider the SAR of a dilute assembly of fractal clusters consisting of spherical magnetite nanoparticles. In accordance with experimental data [8,11] the saturation magnetization of a perfect magnetite nanoparticle is assumed to be *M*_s_ = 450 emu/cm^3^, the magnetic anisotropy constant being [5] *K* = −10^5^ erg/cm^3^. Bearing in mind that the single-domain diameter of spherical magnetite nanoparticle equals *D*_c_ = 64 nm [35], the calculations are performed for nanoparticles in the range of diameters *D* = 20–60 nm. The clusters of nanoparticles are assumed to be tightly bound to surrounding media so that the nanoparticles cannot rotate as a whole under the influence of an alternating magnetic field. The ambient temperature of the media is *T* = 300 K. It is well known that magnetic nanoparticles are usually covered by thin nonmagnetic shells to protect them from oxidation [3]. It was theoretically shown [22] that the intensity of the magneto–dipole interaction in dense clusters of uniaxial nanoparticles depends appreciably on the thickness of the nonmagnetic shell at the nanoparticle surface. To study this effect in an assembly of nanoparticles with cubic and combined anisotropy, in this paper, the calculations are carried out for different nonmagnetic shell thicknesses, *t*_sh_ = 10–30 nm, comparable with the particle diameters.

**Figure 2:**
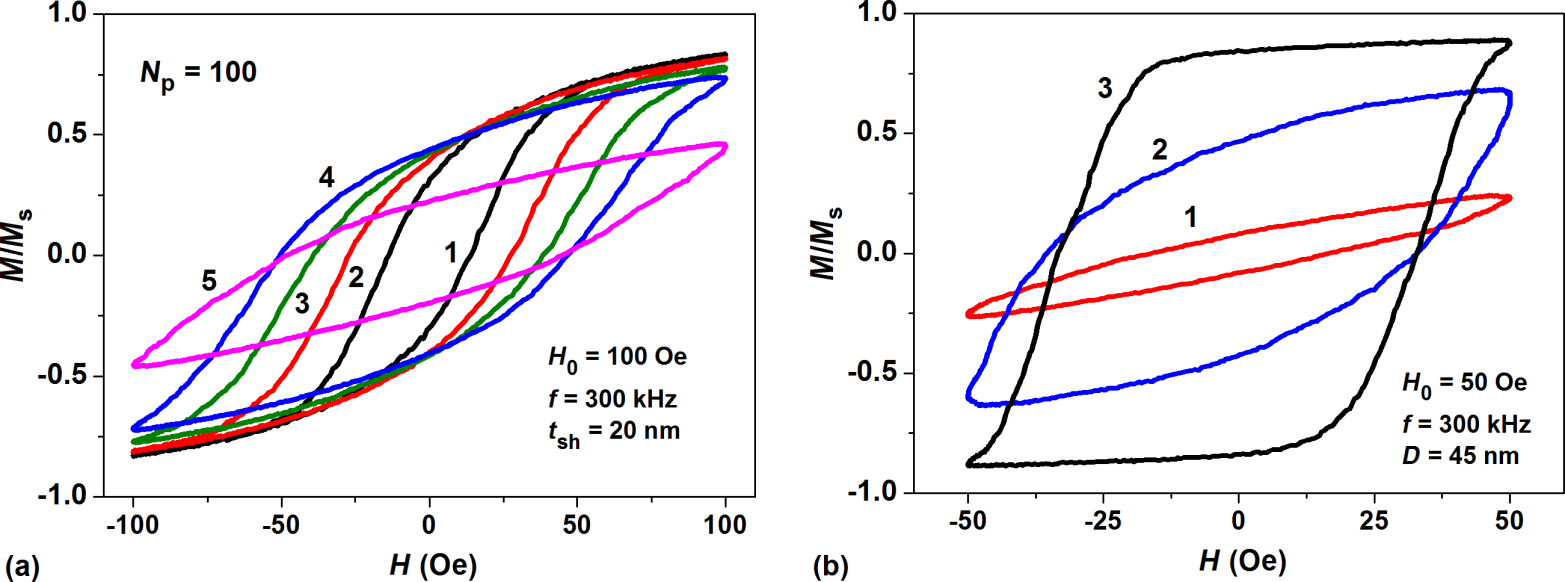
a) Low frequency hysteresis loops of dilute clusters of spherical magnetite nanoparticles with cubic anisotropy and nonmagnetic shell thickness *t*_sh_ = 20 nm for various particle diameters: 1) *D* = 30 nm, 2) *D* = 35 nm, 3) *D* = 40 nm, 4) *D* = 45 nm, 5) *D* = 55 nm; b) hysteresis loop evolution as a function of nonmagnetic shell thickness at the fixed particle diameter *D* = 45 nm: 1) *t*_sh_ = 20 nm, 2) *t*_sh_ = 30 nm, 3) assembly of noninteracting nanoparticles.

Figure 2a shows low frequency hysteresis loops of a dilute assembly of fractal clusters of spherical magnetite nanoparticles with cubic anisotropy as a function of particle diameter for a fixed thickness of the nonmagnetic shells at the particle surfaces, *t*_sh_ = 20 nm. The number of the nanoparticles within the cluster is fixed at *N*_p_ = 100. Figure 2b shows the low frequency hysteresis loops for different thicknesses of nonmagnetic shell at a fixed nanoparticle diameter *D* = 45 nm. The hysteresis loops presented in Figure 2 are averaged over 30 independent cluster realizations.

As Figure 2a shows, the shape and area of the low frequency hysteresis loop of the assembly changes considerably as a function of nanoparticle diameter at fixed values of other parameters. For the case of nanoparticles with *t*_sh_ = 20 nm, the area of the hysteresis loop is maximal for nanoparticles with diameter *D* = 45 nm. Therefore, for assemblies of interacting iron oxide nanoparticles with cubic anisotropy, the optimal particle diameter is considerably larger than that for an assembly of uniaxial nanoparticles [22]. This is because for a particle with cubic (or combined) magnetic anisotropy, the height of the energy barrier between various potential wells is much lower than that for a uniaxial nanoparticle of the same volume [5].

As Figure 2b shows that the optimal diameter of the nanoparticles depends also on the nonmagnetic shell thickness. By increasing the shell thickness one can decrease the intensity of the magneto–dipole interaction among the closest nanoparticles of the cluster. As a result, the area of the hysteresis loop increases as a function of *t*_sh_. One can see in Figure 2b that the area of the hysteresis loop is the largest one for the assembly of noninteracting nanoparticles when formally *t*_sh_ → ∞. On the other hand, the hysteresis loop area diminishes rapidly with the decrease of the nonmagnetic shell thickness, *t*_sh_, so that the SAR of the assembly is very small for *t*_sh_ = 1–5 nm. A similar effect was also observed for interacting assemblies of nanoparticles with uniaxial magnetic anisotropy [22].

**Figure 3:**
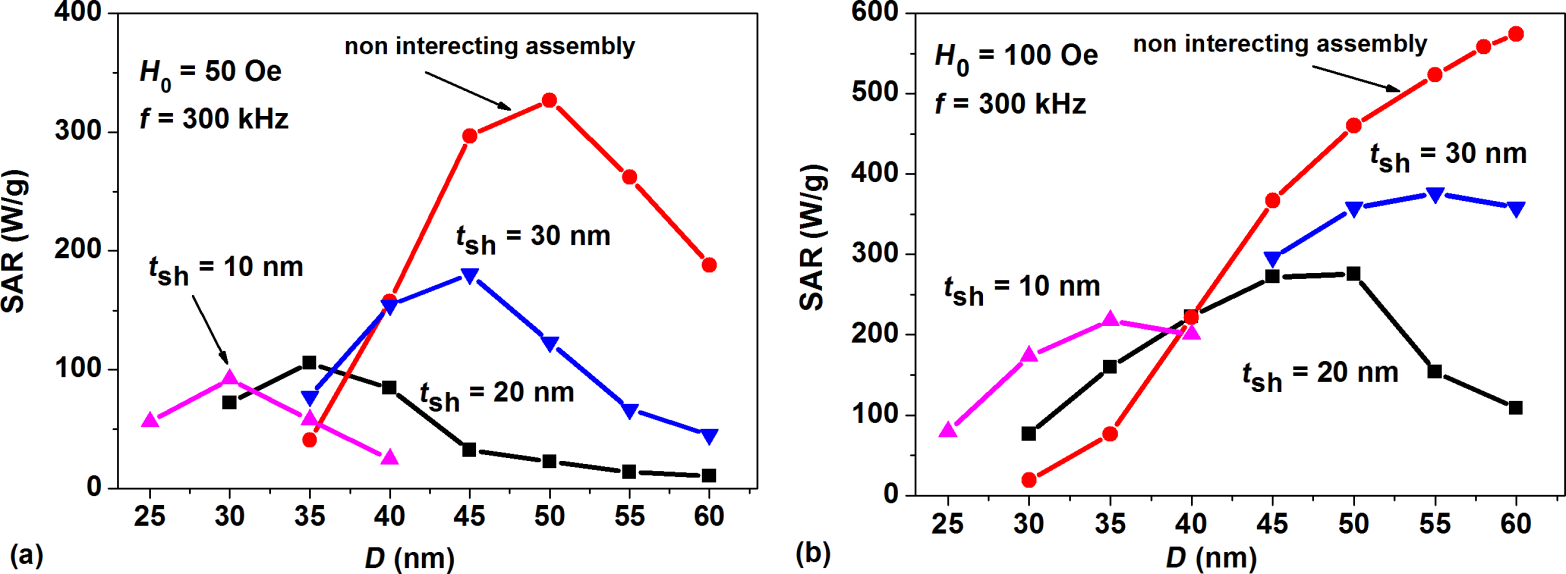
SAR of dilute assemblies of fractal clusters of spherical magnetite nanoparticles with cubic anisotropy as a function of particle diameter at various nonmagnetic shell thicknesses *t*_sh_ and different amplitudes of the alternating magnetic field: a) *H*_0_ = 50 Oe, b) *H*_0_ = 100 Oe.

Figure 3 shows the results of detailed calculations of the SAR for dilute assemblies of clusters of spherical magnetite nanoparticles. The SAR of the assemblies is calculated [3,15] as SAR = 10^−7^ *M*_s_*fA*/ρ (W/g), where *A* is the hysteresis loop area in the variables (*M*/*M*_s_, *H*), and ρ = 5 g/cm^3^ is the density of the magnetite nanoparticles. In accordance with the above arguments, the maximal SAR values correspond to nanoparticles with the largest nonmagnetic shell thickness considered, *t*_sh_ = 30 nm. Besides, the appreciable dependence of SAR on the nanoparticle diameter is revealed in all cases investigated. For comparison, the SAR of noninteracting assemblies of spherical magnetite nanoparticles is also shown in Figure 3a and Figure 3b. Evidently, due to the influence of the strong magneto–dipole interaction in fractal clusters, the SAR of the assembly of clusters decreases considerably with respect to that of an assembly of noninteracting nanoparticles.

One can see in Figure 3a that for a magnetic field of moderate amplitude, *H*_0_ = 50 Oe, the SAR of the assembly has a maximum for particles with diameter *D* = 50 nm. It is well known that the height of the effective energy barrier increases exponentially as a function of particle diameter. For small particle diameters, the effective energy barrier is too small at room temperature. Therefore, the alternating magnetic field has little influence on the assembly behavior. For large particle diameters the barriers are too large, so that the magnetization reversal of the particle is impossible or less probable under an alternating magnetic field of moderate amplitude. However, the probability for particle magnetization reversal increases with the increase in magnetic field amplitude [15]. As Figure 3b shows, in the case *H*_0_ = 100 Oe, the probability of the magnetization reversal is sufficiently high even for nanoparticles with diameters *D* > 50 nm. One can expect that in the case *H*_0_ = 100 Oe the assembly SAR will decrease in the range of diameters *D* > 60 nm. However, the single domain diameter for spherical magnetite nanoparticles equals *D*_0_ = 64 nm [35], so that there are no single-domain magnetite nanoparticles with diameter *D* ≥ *D*_0_.

The results of the numerical simulation shown in Figure 2 and Figure 3 correspond to spherical magnetite nanoparticles. They are also qualitatively true for perfect magnetite nanoparticles of cubic external shape [8,10] having cubic magnetic anisotropy. Actually, it follows from the Brown–Morrish theorem [36] that a single-domain nanoparticle of cubic shape has equal demagnetizing factors. Therefore, its magnetic properties in the first approximation are equivalent to that of a sphere.

It is important to note, however, that the measurement of the angle dependence of the ferromagnetic resonance absorption indicates that the magnetite nanoparticles used in the experiment [13] possess not cubic, but combined or effective uniaxial anisotropy. This behavior may be a consequence of random deviations of the nanoparticle shapes. It was recently shown [23] that for magnetic nanoparticles of soft magnetic type with cubic magneto-crystalline anisotropy, even relatively small perturbations of the spherical shape lead to an appreciable shape magnetic anisotropy. As a result, such nanoparticles possess combined or even effective uniaxial anisotropy. According to the Brown–Morrish theorem [36], the magnetostatic properties of a single domain nanoparticle (i.e., nearly uniformly magnetized particle) of arbitrary shape are equivalent to that of uniformly magnetized ellipsoid with properly chosen semiaxes. For small shape deviations, the semiaxis ratio of an equivalent ellipsoid (for simplicity, spheroid) is close to unity. Therefore, without loss of generality, in the present manuscript, the actual shape of the magnetite nanoparticles is assumed to be spheroid with the principle semiaxis ratio *a*/*b* > 1. To be compared with the case of a spherical nanoparticle, the transverse semiaxis is set to the radius of sphere, *b* = *D*/2, whereas the ratio 2*a*/*D* is a random variable uniformly distributed within a small interval 1 ≤ 2*a*/*D* ≤ ξ_max_, the maximal particle elongation being ξ_max_ ≤ 1.2. It is worth noting that the average particle elongation equals <2*a*/*D*> = 0.5 + ξ_max_/2.

**Figure 4:**
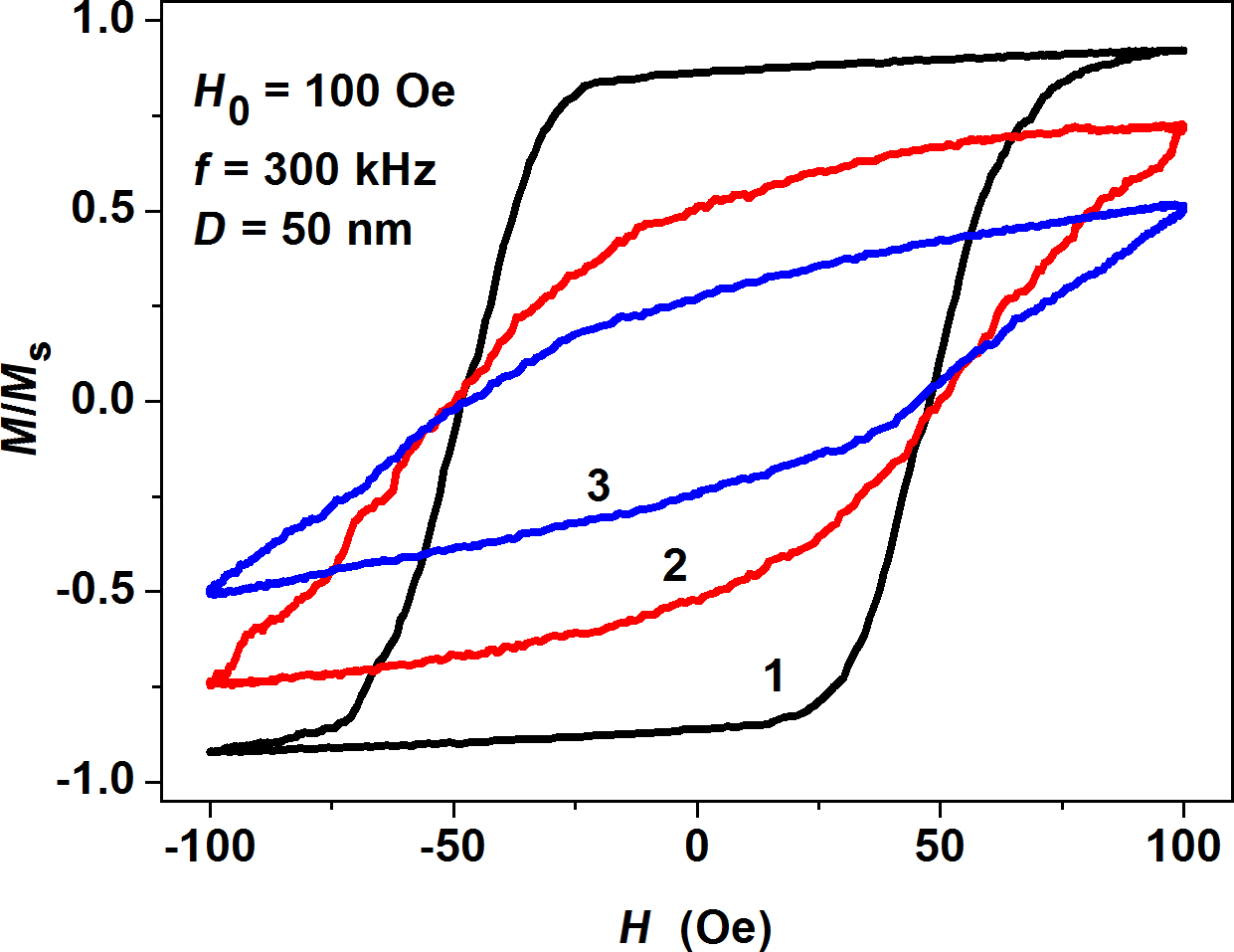
Influence of particle elongation on the hysteresis loop shape for assemblies of noninteracting magnetite nanoparticles: 1) spherical magnetite nanoparticles, 2) magnetite nanoparticles with average elongation <2*a*/*D*> = 1.05, 3) nanoparticles with average elongation <2*a*/*D*> = 1.1.

The effect of the shape deviation is illustrated in Figure 4, where the low frequency hysteresis loops are presented for randomly oriented assemblies of noninteracting magnetite nanoparticles with combined anisotropy, i.e., cubic magneto-crystalline anisotropy plus shape anisotropy due to small elongations of initially spherical nanoparticles along an arbitrary direction with respect to the cubic anisotropy axes. As Figure 4 shows, for an assembly of noninteracting nanoparticles, even small spheroidal shape perturbations lead to appreciable changes of the low frequency hysteresis loop area. The area of the hysteresis loop in Figure 4 decreases as a function of average particle elongation because the shape anisotropy energy increases the effective energy barrier. Thus the probability of the magnetization reversal at a fixed value of the magnetic field amplitude decreases. This effect deserves special consideration for assemblies of interacting magnetite nanoparticles.

### Nanoparticles with combined anisotropy

**Figure 5:**
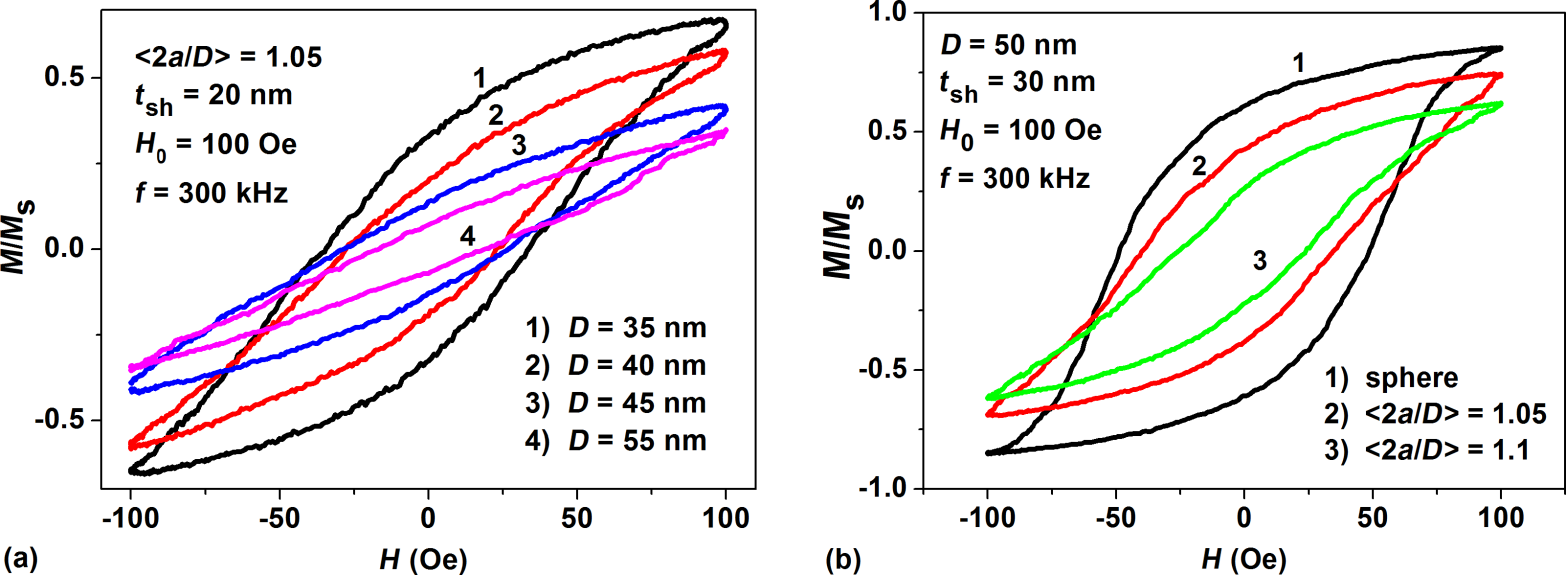
a) Evolution of the hysteresis loops of fractal clusters of magnetite nanoparticles with small average elongation <2*a*/*D*> = 1.05 as a function of transverse particle diameter *D*; b) the dependence of hysteresis loop on the average particle elongation for nanoparticles with fixed transverse diameter, *D* = 50 nm.

To study the influence of the shape anisotropy contribution on the assembly behavior, detailed calculations of the low frequency hysteresis loops have been carried out for dilute assemblies of fractal clusters of magnetite nanoparticles with small random spheroidal perturbations. Figure 5a shows the evolution of the low frequency hysteresis loops of clusters of magnetite nanoparticles with small average elongation <2*a*/*D*> = 1.05 as a function of transverse particle diameter *D*. In Figure 5b we compare the hysteresis loops of clusters consisting of nanoparticles of the same transverse diameter *D* = 50 nm and nonmagnetic shell thickness *t*_sh_ = 30 nm, but having various average elongations. As Figure 5b shows, the area of the hysteresis loop decreases with increase of average nanoparticle elongation due to increase of the shape anisotropy of the elongated magnetite nanoparticles. The largest hysteresis loop area (curve 1 in Figure 5b) corresponds to spherical magnetite nanoparticles with cubic anisotropy.

**Figure 6:**
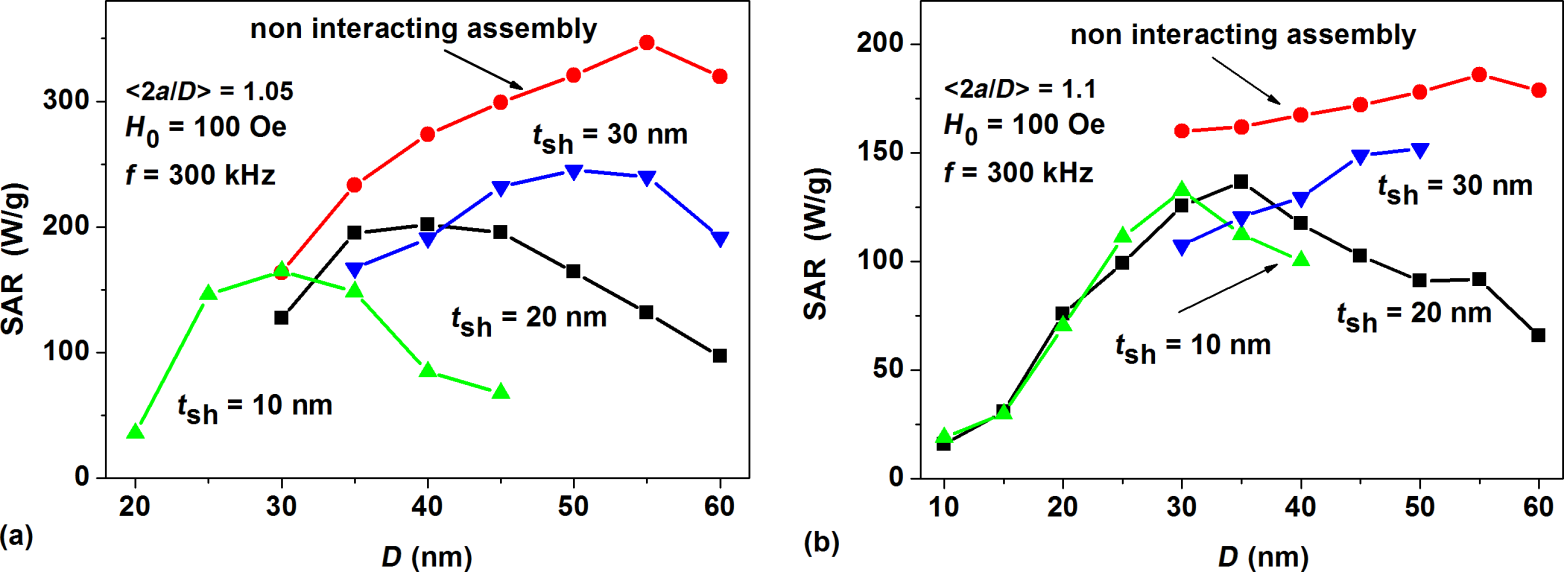
SAR as a function of the transverse nanoparticle diameter *D* for dilute assemblies of fractal clusters of magnetite nanoparticles with various average elongation: a) <2*a*/*D*> = 1.05; b) <2*a*/*D*> = 1.1.

Figure 6 shows the SAR of dilute assemblies of fractal clusters of magnetite nanoparticles with various average elongation and different nonmagnetic shell thickness as a function of transverse particle diameter.

The SAR of the corresponding noninteracting assemblies is also shown in Figure 6a and 6b for comparison. It can be seen that the increase of the nonmagnetic shell thickness leads to an appreciable increase in the maximal SAR value. Simultaneously, the optimal particle diameter increases.

**Figure 7:**
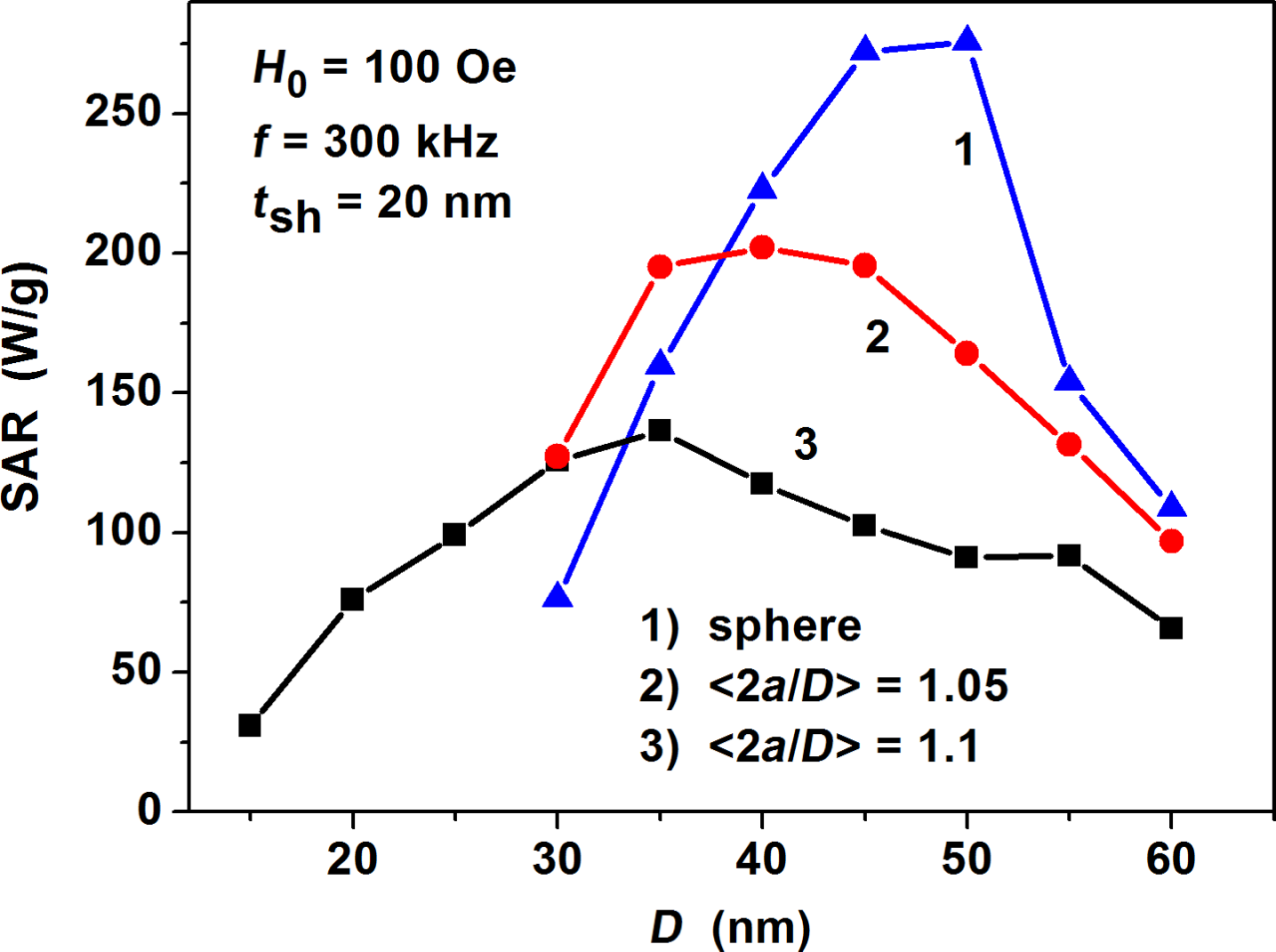
Comparison of the SAR of dilute assemblies of fractal clusters of magnetite nanoparticles with various average elongations, <2*a*/*D*>, but with the same nonmagnetic shell thickness *t*_sh_ = 20 nm.

In Figure 7 we compare the SAR of magnetite nanoparticles with the same nonmagnetic shell thickness, *t*_sh_ = 20 nm, but having various average elongations. One can see that at fixed parameters of the alternating magnetic field, the highest SAR value corresponds to an assembly of fractal clusters of spherical magnetite nanoparticles.

## Discussion

It has been recently shown [22] that for an assembly of interacting magnetic nanoparticles with uniaxial magnetic anisotropy there is an optimal particle diameter when the SAR of the assembly is maximal. According to Figure 3 and Figure 6, this is also true for an assembly of magnetite nanoparticles with cubic or combined anisotropy. Unfortunately, this important fact attracts little interest of researchers working in the field of magnetic hyperthermia. Actually, different methods of chemical synthesis of iron oxide nanoparticles give particles of various characteristic diameters [3] that are usually far from the optimal theoretical values. Another important issue is the nature of the magnetic anisotropy of individual nanoparticles of the assembly. Are the magnetic nanoparticles single crystals or do they have a mostly polycrystalline structure? What is the influence of the shape anisotropy on the particle behavior? The knowledge of the actual experimental situation is necessary to predict the heating ability of a real assembly of magnetic nanoparticles.

It has been recently stressed [10] that in most of the experiments [7–8,11–12] the SAR measurements were performed for assemblies of nanoparticles distributed in a viscous liquid, although the ultimate goal of the investigations related to magnetic hyperthermia is to study the heating ability of magnetic nanoparticles distributed in tumor cells. This point is important, because the SAR of the assembly distributed in liquid can be substantially higher than that for an assembly of the same nanoparticles distributed in a biological medium [10]. This is because the nanoparticles in liquid can be well separated, so that the influence of the magneto–dipole interaction on the assembly behavior is not significant. In addition, isolated nanoparticles can freely rotate in a viscous fluid as a whole under the action of an alternating external magnetic field. This effect gives an additional contribution to the assembly SAR in the alternating magnetic field [3,16]. On the contrary, in biological media, nanoparticles have a tendency to aggregate because of active biological processes, such as cellular internalization in endocytic compartments [10,13]. As a result, the nanoparticles in biological media may form fractal clusters [24–25] with strong magneto–dipole interaction between the nanoparticles of the cluster. Clusters of the nanoparticles are tightly bound to surrounding tissue, so that the rotation of the nanoparticles of the assembly is restricted. In the present work, these important factors are taken into account when calculating the SAR of dense assemblies of nanoparticles distributed in a biological medium. Furthermore, it is also shown that the SAR of dense assemblies of magnetic nanoparticles can be substantially increased if the nanoparticles are covered with sufficiently thick nonmagnetic shells, because the presence of shells decreases the intensity of the magneto–dipole interaction in the assembly. This effect deserves experimental confirmation.

It is well known that the assembly SAR, as a rule, increases with increasing frequency *f* and amplitude *H*_0_ of the alternating magnetic field. However, for medical reasons, the product of these parameters should be restricted by a therapeutic limit, *f* × *H*_0_ ≤ 5 × 10^9^ a/m/s [2–3]. Under this condition the alternating magnetic field is safe for healthy body tissues. Worth noting is that very large SAR values reported in a number of experiments [7–8,11] were obtained at sufficiently high frequencies, *f* > 500 kHz, and large magnetic field amplitudes, *H*_0_ = 300 Oe, when the product *f* × *H*_0_ exceeds the therapeutic limit considerably. Furthermore, as we mentioned above, these experiments were performed for dilute assemblies of nanoparticles distributed in a viscous liquid, i.e., under the conditions far from that of magnetic hyperthermia.

Instead, in this paper, we show that for assemblies of magnetite nanoparticles with cubic or combined magnetic anisotropy with optimal choice of nanoparticle size and nonmagnetic shell thickness one can obtain rather large SAR values, on the order of 200–250 W/g, at a characteristic frequency *f* = 300 kHz and a moderate magnetic field amplitude *H*_0_ = 100 Oe. These SAR values correspond to assemblies of interacting magnetite nanoparticles distributed in a biological medium. The use of moderate magnetic field amplitude values seems important, as the creation of alternating magnetic fields of large amplitude, *H*_0_ = 200–300 Oe, requires generation of strong alternating electric currents, which is expensive and can be dangerous in a clinical treatment. This makes an assembly of magnetite nanoparticles of optimal size having cubic or combined magnetic anisotropy very promising for application in magnetic hyperthermia.

## Conclusion

In this paper the low-frequency hysteresis loops in alternating magnetic field have been calculated for interacting assemblies of magnetite nanoparticles with cubic and combined anisotropy. The magnetite nanoparticles are widely used in biomedical research [3–14]. However, when analyzing experimental data, it is implicitly assumed that they have a uniaxial type of magnetic anisotropy, though a single crystal of magnetite possesses cubic type magnetic anisotropy. In fact, perfect magnetite nanoparticles of spherical shape should have cubic type magnetic anisotropy. On the other hand, if the nanoparticle shape deviates from that of a sphere, anisotropy energy appears. The shape anisotropy contribution can be appreciable even for slightly elongated nanoparticles of soft magnetic type [23]. In this case, the magnetite nanoparticles possess combined magnetic anisotropy. This important fact has to be taken into account to understand the behavior of assemblies of magnetite nanoparticles in an applied alternating magnetic field.

However, an adequate description of the nature of the magnetic anisotropy of magnetite nanoparticles is not sufficient to carry out reliable SAR calculations for an assembly of these nanoparticles distributed in biological media. One has to also take into account the influence of the strong magneto–dipole interaction on the behavior of fractal clusters of nanoparticles arising often within the biological cells or in the intracellular space [13,24–25]. This can be done using the stochastic LL equation [22,28–31] that describes the dynamics of the particle magnetic moments taking into account both the magneto–dipole interactions and the effect of thermal fluctuations.

For an assembly of superparamagnetic nanoparticles with uniaxial anisotropy, it has been recently shown [22] that the existence of nonmagnetic shells of appreciable thickness at the nanoparticle surface considerably reduces the intensity of the magneto–dipole interaction within the cluster. In addition, a substantial dependence of the assembly SAR on the mean nanoparticle diameter has been revealed. Using the same approach, detailed calculations of the SAR for assemblies of interacting magnetite nanoparticles have been carried out in this paper. For assemblies of spherical magnetite nanoparticles with cubic magnetic anisotropy under moderate magnetic field amplitudes *H*_0_ = 50–100 Oe and frequency *f* = 300 kHz, the optimal particle diameters are shown to be within the range *D* = 45–55 nm, depending on the nonmagnetic shell thickness at the particle surface. The maximal SAR = 350 W/g is obtained at *H*_0_ = 100 Oe and *t*_sh_ = 30 nm. The low frequency hysteresis loops were also calculated for assemblies of slightly elongated magnetite nanoparticles with a small average elongation, <2*a*/*D*> = 1.05–1.1, having combined magnetic anisotropy. It is found that due to the influence of the shape anisotropy contribution, the maximal SAR of assemblies of magnetite nanoparticles with combined anisotropy decreases to 150–250 W/g depending on the average particle elongation. It is worth mentioning that rather high SAR values, on the order of 250–350 W/g, have been obtained for low values of magnetic field amplitudes, *H*_0_ = 50–100 Oe. Therefore, the assemblies of magnetite nanoparticles with cubic or combined anisotropy seem promising for application in magnetic nanoparticle hyperthermia.
